# Supplementary material for: Environmental Regulation and Gene-by-Environment Interaction Influence RAP1 Activity and its Impact on Gene Expression
Source: bioRxiv. 2026 May 9:2026.05.06.723246. Preprint. [Version 1] doi: 10.64898/2026.05.06.723246 (PMC13174685; doi:10.64898/2026.05.06.723246)
Supplement: Supplement 1 [file NIHPP2026.05.06.723246v1-supplement-1.pdf]

Supplementary

S-Figure1: Overlap of shared GO enriched terms in different comparisons (HS vs YPD, YPAC vs YPD, and YPAC vs HS) at maximum *RAP1* levels**.**

S-Figure2: Overlap of shared GO enriched terms at all *RAP1* levels in YPD, HS and YPAC environment.

Table Legends

Supplementary

S-Table1: Gene expression (FPKM) values across various *RAP1* levels in YPD, HS and YPAC environment.

S-Table2: List of overlapping significant DEGs at max *RAP1* level

S-Table3: List of Heat and Acetate genes downloaded from SGD used for GSEA

S-Table4: Gene ontology enrichment terms observed at max *RAP1* levels in YPD, HS and YPAC

S-Table5: Gene ontology enrichment terms observed at various *RAP1* levels in YPD, HS and YPAC

S-Table6: Gene ontology enrichment terms for genes belonging to different network modules.

S-Table7: Summary of sample collection, RNA sequencing depth and mapping percentages.
